# Supplementary material for: Relational Coordination at the Primary–Secondary Care Interface: Insights from a Cross-Sectional Survey in the South Tyrolean Healthcare System
Source: Int J Environ Res Public Health. 2024 Mar 30;21(4):425. doi: 10.3390/ijerph21040425 (PMC11050390; doi:10.3390/ijerph21040425)
Supplement: Supplementary file 1 [file ijerph-21-00425-s001.zip › ijerph-2915897-supplementary.pdf]

Table S1. Relational coordination scores among healthcare professional groups by dimensions.

		Ratings of [1]					
		GPs	Hospital physicians	Administrative personnel	Nurses	External	Overall
		Frequency of communication					
	GPs	3.65	3.04	2.45	3.61	3.19	3.65
	Hospital physicians	2.86	3.93	3.15	2.52	3.12	3.93
	Administrators	2.14	2.36	3.07	2.21	2.45	3.07
	Nurses	3.71	3.04	3.42	4.09	3.57	4.09
	Total	3.28	3.41	3.14	3.28	3.28	3.85
	Total (weighted)	3.05	3.55	3.09	2.90	3.15	3.92
		Timeliness of communication					
	GPs	4.28	2.68	2.98	4.11	3.51	4.28
	Hospital physicians	2.98	3.91	3.12	3.26	3.32	3.91
	Administrators	3.21	3.21	3.36	3.5	3.32	3.36
	Nurses	3.12	2.93	3.7	4.32	3.52	4.32
	Total	3.24	3.33	3.32	3.80	3.42	3.98
	Total (weighted)	3.20	3.53	3.21	3.56	3.38	4.11
		Accuracy of communication					
	GPs	4.05	3.65	3.1	3.9	3.67	4.05
	Hospital physicians	3.02	3.85	3.09	3.45	3.35	3.85
	Administrators	3.93	3.64	3.43	3.50	3.63	3.43
	Nurses	3.27	3.34	3.77	4.26	3.66	4.26
	Total	3.30	3.62	3.36	3.83	3.53	3.91
	Total (weighted)	3.27	3.73	3.22	3.64	3.47	4.03
		Problem-solving communication					
	GPs	4.27	3.51	3.06	4.21	3.76	4.27
	Hospital physicians	3.52	3.90	3.22	3.70	3.58	3.9
Ratings by [1]	Administrators	3.43	3.43	3.29	3.43	3.39	3.29
	Nurses	3.44	3.41	3.70	4.20	3.69	4.20
	Total	3.60	3.64	3.38	3.96	3.65	3.95
	Total (weighted)	3.61	3.73	3.28	3.83	3.61	4.05
		Shared goals					
	GPs	4.09	3.30	2.61	4.02	3.55	4.09
	Hospital physicians	3.18	3.81	2.84	3.36	3.40	3.81
	Administrators	2.86	3.29	3.07	3.14	3.29	3.07
	Nurses	3.40	3.23	3.32	4.2	3.55	4.20
	Total	3.40	3.50	2.99	3.78	3.48	3.86
	Total (weighted)	3.32	3.61	2.90	3.57	3.44	3.98
		Shared knowledge					
	GPs	4.40	3.45	2.89	3.66	3.60	4.40

Hospital physicians	3.27	3.83	2.94	3.13	3.29	3.83
Administrators	2.50	2.50	2.71	2.43	2.54	2.71
Nurses	3.38	3.30	3.17	4.28	3.53	4.28
Total	3.47	3.54	3.01	3.63	3.41	3.9
Total (weighted)	3.39	3.59	2.95	3.33	3.32	4.06
Mutual respect						
GPs	4.46	3.44	3.39	4.24	3.88	4.46
Hospital physicians	3.94	4.17	3.73	4.05	3.97	4.17
Administrators	3.71	3.93	3.64	3.93	3.8	3.64
Nurses	3.79	3.63	3.98	4.42	3.96	4.42
Total	3.96	3.85	3.77	4.22	3.95	3.92
Total (weighted)	3.97	3.97	3.71	4.13	3.94	4.11

[1] Relational coordination within workgroups scoring: weak < 4.1, moderate 4.1–4.6, and strong > 4.6; between workgroup scoring: weak < 3.5, moderate 3.5–4.0. and strong > 4.0 [19]. Abbreviations: GPs, General Practitioners.

Table S2. Correlations between demographic, professional, and feedback factors with relational coordination scores.

RC	Age[1]	Years in service[1]	GPs' compliance with referral criteria[1]	GPs receiving referral feedback[1]	Hospital physicians' perception of inappropriate referral priority[1]	Hospital physicians giving feedback on inappropriate referrals[1]	Hospital physicians' rating of the quality of clinical questions posed by GPs[1]
Overall	n.s.	n.s.	n.s.	n.s.	0.255***	n.s.	-0.306***
Within group	n.s.	n.s.	-0.238**	n.s.	n.s.	n.s.	n.s.
Between groups	n.s.	n.s.	n.s.	n.s.	-0.244***	n.s.	-0.324***
GPs	n.s.	n.s.	-0.238***	n.s.	-0.270***	n.s.	-0.389***
Hospital physicians	n.s.	n.s.	n.s.	n.s.	n.s.	n.s.	n.s.
Administrators	n.s.	n.s.	n.s.	n.s.	-0.185***	n.s.	-0.188***
Nurses	n.s.	n.s.	n.s.	n.s.	-0.140**	n.s.	-0.176**
Frequency of communication	0.116**	n.s.	n.s.	n.s.	-0.134*	-0.126*	n.s.
Timeliness of communication	n.s.	n.s.	n.s.	n.s.	-0.150**	n.s.	-0.288***
Accuracy of information	n.s.	n.s.	n.s.	n.s.	-0.200***	n.s.	-0.297***
Problem-solving communication	n.s.	n.s.	n.s.	n.s.	-0.229***	n.s.	-0.234***

Shared goal	n.s.	n.s.	n.s.	n.s.	-0.228***	n.s.	-0.272***
Shared knowledge	n.s.	n.s.	-0.191*	n.s.	-0.108*	n.s.	n.s.
Mutual respect	n.s.	n.s.	n.s.	n.s.	-0.193***	n.s.	-0.244***

[1]Spearman correlation coefficients were utilized to determine the significance of relationships between various demographic characteristics, professional factors, and feedback practices and relational coordination (RC) scores. Significance levels are denoted by asterisks, where * $p < 0.05$, ** $p < 0.01$, and *** $p < 0.001$. The RC scores are divided into internal coordination (RC within group), external coordination (RC between groups), and specific professional groups: general practitioners (GP), hospital physicians, administrative personnel, and nurses. Additional dimensions of RC are included: frequency, timeliness and accuracy of information, problem-solving communication, shared goals, shared knowledge, and mutual respect. Negative correlations suggest that higher values in the demographic or professional factor are associated with lower perceived RC, while positive correlations suggest higher RC. Additional abbreviations: n.s., not significant; n.d., no data.

File S1

Questionnaire

Questions for general practitioners

Part 1—Relational Coordination Survey

1. How often do you communicate with hospital doctors? a) Never b) Rarely c) Sometimes d) Frequently e) Always
2. How frequently do you communicate with administrative staff? a) Never b) Rarely c) Sometimes d) Frequently e) Always
3. How frequently do you communicate with the Sprengel nursing staff? a) Never b) Rarely c) Sometimes d) Frequently e) Always
4. How timely is communication with hospital doctors? a) Very delayed b) Delayed c) Average d) Timely e) Very timely
5. How timely is communication with administrative staff? a) Very delayed b) Delayed c) Average d) Timely e) Very timely
6. How timely is communication with the Sprengel nursing staff? a) Very delayed b) Delayed c) Average d) Timely e) Very timely
7. How accurate is the information you receive from hospital doctors? a) Not at all accurate b) Slightly accurate c) Averagely accurate d) Fairly accurate e) Very accurate
8. How accurate is the information you receive from administrative staff? a) Not at all accurate b) Slightly accurate c) Averagely accurate d) Fairly accurate e) Very accurate
9. How accurate is the information you receive from the Sprengel nursing staff? a) Not exactly b) Not very precise c) Average accuracy d) Pretty accurate e) Very precise
10. To what extent is communication with hospital doctors solution oriented (i.e., focused on solving problems rather than blaming)? a) Not at all solution oriented b) Slightly solution oriented c) Averagely solution oriented d) Fairly solution oriented e) Very solution oriented
11. To what extent is communication with administrative staff solution oriented (i.e., focused on solving problems rather than blaming)? a) Not at all solution oriented b) Slightly solution oriented c) Averagely solution oriented d) Fairly solution oriented e) Very solution oriented
12. To what extent is the communication with nursing staff solution oriented (i.e., focused on solving problems rather than placing blame)? a) Not solution oriented at all b) Not very solution oriented c) Averagely solution oriented d) Fairly solution oriented e) Very solution oriented
13. To what extent do you share common goals with hospital doctors? a) Not at all b) A little c) Partly/Sometimes d) Quite a bit e) Completely
14. To what extent do you share common goals with the administrative staff? a) Not at all b) A little c) Partly/Sometimes d) Quite a bit e) Completely
15. To what extent do you share common goals with the Sprengel nursing staff? a) Not at all b) A little c) Partly/Sometimes d) Quite a bit e) Completely
16. To what extent do you have common knowledge about the respective tasks and responsibilities of hospital doctors? a) Not at all b) A little c) Partly/Sometimes d) Quite a bit e) Completely

17. To what extent do you have common knowledge about the respective tasks and responsibilities of the administrative staff? a) Not at all b) A little c) Partly/Sometimes d) Quite a bit e) Completely
18. To what extent do you have common knowledge about the respective tasks and responsibilities of the Sprengel nursing staff? a) Not at all b) A little c) Partly/Sometimes d) Quite a bit e) Completely
19. To what extent do you have mutual respect in your collaboration with hospital doctors? a) Not at all b) A little c) Partly/Sometimes d) Quite a bit e) Completely
20. To what extent do you have mutual respect in your cooperation with the administrative staff? a) Not at all b) A little c) Partly/Sometimes d) Quite a bit e) Completely
21. To what extent do you have mutual respect in your collaboration with the Sprengel nursing staff? a) Not at all b) A little c) Partly/Sometimes d) Quite a bit e) Completely

Part 2—Job Satisfaction and Intention to Stay

22. Overall, how satisfied are you with your current job? a) Very dissatisfied b) Dissatisfied c) Averagely satisfied d) Fairly satisfied) Completely satisfied
23. "I plan to leave the South Tyrolean medical company/the convention with the country as soon as possible". a) Completely agree b) Somewhat agree c) Neither agree nor agree d) Somewhat disagree e) Strongly disagree to

Part 3—Working Conditions

24. How satisfied are you generally with the collaboration and communication between you and the hospital doctors? a) Very satisfied b) Satisfied c) Dissatisfied d) Very dissatisfied
25. How satisfied are you generally with the collaboration and communication between you and the Sprengel nursing staff? a) Very satisfied b) Satisfied c) Dissatisfied d) Very dissatisfied
26. If anything is unclear, can you reach your colleagues in the hospital by phone? a) Often b) Occasionally c) Rarely d) Never
27. If anything is unclear, can you contact the Sprengel nursing staff by telephone? a) Often b) Occasionally c) Rarely d) Never
28. How often do you contact hospital colleagues about patients via email? a) Frequently b) Occasionally c) Rarely d) Never
29. How often do you contact the Sprengel nursing staff about patients via email? a) Frequently b) Occasionally c) Rarely d) Never
30. In your opinion, how often do you manage to avoid a referral to a specialist by making a phone call or shortening the time it takes to reach your colleague? a) Often b) Occasionally c) Rarely d) Never
31. How often do you estimate that you refer your patients to a hospital doctor? a) In 80% of (re-)acute counseling situations b) In 50% of (re-)acute counseling situations c) In 30% of (re-)acute counseling situations d) In 10% of (re-)acute counseling situations
32. Which of the factors in the majority of cases determines your decision to refer a patient to a specialist? a) Severity of symptoms b) Urgency of treatment c) Unclear diagnosis d) Patient's wishes e) Time pressure in your practice f) Other… (free text)
33. In your opinion, how did the "homogeneous waiting group" (HWG) criteria influence the allocations? a) Relieved b) more difficult c) shortened waiting times d) waiting times extended e) waiting times are left unaffected
34. How often do you estimate to prescribe priority visits or examinations? a) In less than 5% of referrals b) In 10% of referrals c) In 20% of referrals d) In 30% of referrals e) In … % of referrals (enter number)

35. If you know the criteria or have access to them through your practice program, do you agree with them? a) Yes, completely b) Yes, mostly c) No, not really d) No, not at all
36. How often do you think you can comply with the "homogeneous waiting group" (HWG) criteria for your transfers? a) In 90% of my transfers b) In 70% of my transfers c) In 50% of my transfers d) In less than 30% of my transfers
37. What is the most common reason for non-compliance with the "homogeneous waiting group" (HWG) criteria? a) Waiting times too long for a "normal" (postponable) visit b) Clinical urgency does not meet HWG criteria c) Complaints do not respond to conventional therapy d) Unclear picture that is not listed under the HWG criteria e) Pressure and/or fear from patients or relatives f) Other: …
38. Which subjects/areas require more changes/adjustments in terms of priority criteria? (open question)
39. Are the waiting times specified according to priority (B 10 days, D 30 days) mostly adhered to? a) Yes, completely b) Yes, mostly c) No, not really d) No, not at all
40. If not, in which subjects? (open question)
41. In your opinion, are the waiting times for "normal", deferrable visits generally too long? a) Yes, completely b) Yes, mostly c) No, not really d) No, not at all
42. If, in your opinion, the patient needs a priority (B within 10 days) specialist visit/examination and he only gets it after a significantly longer period of time, how do you proceed? a) You recommend that the patient go to a private specialist or structure b) You try to arrange an earlier appointment directly with a hospital doctor c) You prescribe an urgent visit to the patient and send him to first aid d) You recommend that the patient go directly to first aid without your referral
43. Has the agreement of many private structures with the medical service shortened waiting times? a) Yes, completely b) Yes, mostly c) No, not really d) No, not at all
44. What problems have you encountered with transfers to private, conventional structures? (Multiple answers possible) a) None b) Lower quality of the services provided c) More bureaucratic effort (prescription of operations and follow-up treatment by the family doctor, etc.) d) No follow-up care e) Other
45. Are you in favor of expanding alternative care modalities (e.g., telemedicine)? a) Yes, definitely b) Yes, probably c) No, not likely d) No, not at all
46. Have you received feedback from specialists in the past that a referral was inappropriate? a) Yes, often b) Yes, occasionally c) No, rarely d) No, never
47. Overall, how satisfied are you with the current system of specialist assignments? a) Very satisfied b) Satisfied c) Dissatisfied d) Very dissatisfied
48. How important would it be to you that circulars from the medical company contain a brief summary of the organizational changes? a) Very important b) Important c) Less important d) Unimportant
49. How important would it be to you that the medical company created a link where all the forms, circulars and regulations that affect you as a family doctor can be found? a) Very important b) Important c) Less important d) Unimportant
50. How important would it be to you to introduce a reserved telephone number only for general practitioners and a number only for check-ups? a) Very important b) Important c) Less important d) Unimportant
51. How important would it be to you that the basic medicine services are led by experienced general practitioners? a) Very important b) Important c) Less important d) Unimportant

52. Should 3 to 4 annual meetings be organized with the medical management of the basic medical services at the district level in order to be able to address problems in a timely manner and to discuss proposed solutions? a) Yes, definitely b) Yes, but less often c) No, probably not d) No not at all
53. How important is it for you that every specialist in the hospital has a fixed telephone number where he can be reached every day and guarantees a binding callback if he is not available? a) Very important b) Important c) Less important d) Not important
54. How important is it to you that free slots in the specialist outpatient clinics can be reserved for patients with special questions that can be answered by the family doctor via direct telephone contact? a) Very important b) Important c) Less important d) Not important
44. Do you have any further comments or suggestions for improving collaboration and communication with the hospital's specialist colleagues and the specialist assignment system? (Open question)
45. Do you have any further comments or suggestions for improving collaboration and communication with the Sprengel nursing staff? (Open question)

Part 4—Additional Questions

- Sociodemographic information (age, gender, health district, city/rural or population categories of the place, group practice)
- How many years have you worked as a family doctor?
- How many patients are you currently enrolled in?
- How many patient contacts (excluding repeat medication prescriptions) do you have on average per week?

Questions for specialists

Part 1—Relational Coordination Survey

1. How often do you communicate with general practitioners? a) Never b) Rarely c) Sometimes d) Frequently e) Always
2. How frequently do you communicate with administrative staff? a) Never b) Rarely c) Sometimes d) Frequently e) Always
3. How often do you communicate with hospital doctors from other disciplines? a) Never b) Rarely c) Sometimes d) Frequently e) Always
4. How promptly does communication with general practitioners take place? a) Very delayed b) Delayed c) Average d) Timely e) Very timely
5. How timely is communication with administrative staff? a) Very delayed b) Delayed c) Average d) Timely e) Very timely
6. How timely is communication with hospital doctors from other departments? a) Very delayed b) Delayed c) Average d) Timely e) Very timely
7. How accurate is the information you receive from general practitioners? a) Not at all accurate b) Slightly accurate c) Averagely accurate d) Fairly accurate e) Very accurate
8. How accurate is the information you receive from administrative staff? a) Not at all accurate b) Slightly accurate c) Averagely accurate d) Fairly accurate e) Very accurate
9. How accurate is the information you receive from hospital doctors in other disciplines? a) Not at all accurate b) Slightly accurate c) Averagely accurate d) Fairly accurate e) Very accurate

10. To what extent is communication with general practitioners solution oriented (i.e., focused on solving problems rather than blaming)? a) Not at all solution oriented b) Slightly solution oriented c) Averagely solution oriented d) Fairly solution oriented e) Very solution oriented
11. To what extent is communication with administrative staff solution oriented (i.e., focused on solving problems rather than blaming)? a) Not at all solution oriented b) Slightly solution oriented c) Averagely solution oriented d) Fairly solution oriented e) Very solution oriented
12. To what extent is communication with hospital doctors from other disciplines solution oriented (i.e., focused on solving problems rather than blaming)? a) Not solution oriented at all b) Not very solution oriented c) Averagely solution oriented d) Fairly solution oriented e) Very solution oriented
13. To what extent do you share common goals with general practitioners? a) Not at all b) A little c) Partly/Sometimes d) Quite a bit e) Completely
14. To what extent do you share common goals with the administrative staff? a) Not at all b) A little c) Partly/Sometimes d) Quite a bit e) Completely
15. To what extent do you share common goals with hospital doctors from other disciplines? a) Not at all b) A little c) Partly/Sometimes d) Quite a bit e) Completely
16. To what extent do you have common knowledge about the respective tasks and responsibilities of general practitioners? a) Not at all b) A little c) Partly/Sometimes d) Quite a bit e) Completely
17. To what extent do you have common knowledge about the respective tasks and responsibilities of the administrative staff? a) Not at all b) A little c) Partly/Sometimes d) Quite a bit e) Completely
18. To what extent do you have common knowledge about the respective tasks and responsibilities of hospital doctors from other disciplines? a) Not at all b) A little c) Partly/Sometimes d) Quite a bit e) Completely
19. To what extent do you have mutual respect in your collaboration with general practitioners? a) Not at all b) A little c) Partly/sometimes d) Quite a bit e) Completely
20. To what extent do you have mutual respect in your cooperation with the administrative staff? a) Not at all b) A little c) Partly/Sometimes d) Quite a bit e) Completely
21. To what extent do you have mutual respect in your collaboration with hospital doctors from other disciplines? a) Not at all b) A little c) Partially/sometimes d) Quite a bit e) Completely

Part 2—Job Satisfaction and Intention to Stay

22. Overall, how satisfied are you with your current job? a) Very dissatisfied b) Dissatisfied c) Averagely satisfied d) Fairly satisfied) Completely satisfied
23. "I plan to leave the South Tyrolean medical company/the convention with the country as soon as possible." a) Completely agree b) Somewhat agree c) Neither agree nor agree d) Somewhat disagree e) Strongly disagree to

Part 3—Working Conditions

23. How satisfied are you generally with the collaboration and communication between you and the family doctors? a) Very satisfied b) Satisfied c) Dissatisfied d) Very dissatisfied
24. If anything is unclear, can you reach your family doctor by phone? a) Often b) Occasionally c) Rarely d) Never
25. In your work, how often do you come across patients with a priority or urgent referral from a general practitioner for whom, in your opinion, a “normal”, deferrable visit would have been indicated? a) In

less than 10% of the priority ones or urgent visits b) In 10 to 30% of priority or urgent visits c) In 30 to 60% of priority or urgent visits d) In over 60% of priority or urgent visits

26. In your opinion, which factors most often influence general practitioners' decisions to refer patients to specialists and with what urgency? (Multiple answers possible) a) Severity of symptoms b) Urgency of treatment c) Unclear diagnosis d) Patient wishes e) Time pressure in the practice f) Others (please explain ______________________.)
27. How well informed are you about the current guidelines and "homogeneous waiting group" criteria (HWG) for general practitioner patient allocation priorities? a) Very good b) Good c) Moderate d) Poor
28. What do you think are the most common reasons for inappropriate specialist referrals? Open question

    ____
29. Are you in favor of expanding alternative care modalities (e.g., telemedicine)? a) Yes, definitely b) Yes, probably c) No, not likely d) No, not at all
30. In your opinion, are waiting times for "normal" deferrable visits generally too long? a) Yes, completely b) Yes, mostly c) No, not really d) No, not at all
31. How frequently do you provide feedback to GPs about inappropriate referrals? a) Very common b) Common c) Occasionally d) Rare e) Never
32. Do you think improving the referral priority criteria (HWG) of patients from general practitioners to specialists could help reduce the number of inappropriate referrals? a) Yes, definitely b) Yes, probably c) No, not likely d) No, not at all
33. Overall, how satisfied are you with the current system of specialist assignments? a) Very satisfied b) Satisfied c) Dissatisfied d) Very dissatisfied
34. What do you think could be done to shorten waiting times? Open question

    ____
35. How well do you feel your hospital department is connected to general medicine? a) Very good b) Good c) Bad d) Very bad
36. How important do you think it is to have "a fixed telephone number and/or email address for each department", behind which there is a specialist who can be contacted daily by the general practitioner? a) Very important b) Important c) Unimportant d) Very unimportant
37. How necessary do you think it is to create free slots in specialist outpatient clinics for patients with special questions that can be answered by the family doctor via direct telephone contact? a) Very necessary b) Necessary c) Unnecessary d) Very unnecessary
38. Do you have any further comments or suggestions for improving collaboration and communication with family doctors and the specialist referral system? Open question

    ____
39. How do you assess the current working atmosphere and the collaboration with colleagues from other departments in the hospital? a) Very good b) Good c) Average d) Bad e) Very bad
40. Do you have any further comments or suggestions for improving collaboration with colleagues from other departments in the hospital? Open question ___________________________________
41. How do you rate the information on the clinical questions of general medical referrals? a) Very good b) Good c) Fair d) Poor e) Very bad

42. How often do you feel that patients receive inappropriate specialist referrals based on patient demands? a) Very often b) Frequently c) Occasionally d) Never

Part 4—Additional Questions

- Sociodemographic information (age, gender, hospital)
- How many years (do you work in the hospital, work in the specialty)

Questions for the administrative staff

Part 1—Relational Coordination Survey

1. How often do you communicate with hospital doctors? a) Never b) Rarely c) Sometimes d) Frequently e) Always
2. How often do you communicate with general practitioners? a) Never b) Rarely c) Sometimes d) Frequently e) Always
3. How frequently do you communicate with the Sprengel nursing staff? a) Never b) Rarely c) Sometimes d) Frequently e) Always
4. How timely is communication with hospital doctors? a) Very delayed b) Delayed c) Average d) Timely e) Very timely
5. How timely is communication with general practitioners? a) Very delayed b) Delayed c) Average d) Timely e) Very timely
6. How timely is communication with the Sprengel nursing staff? a) Very delayed b) Delayed c) Average d) Timely e) Very timely
7. How accurate is the information you receive from hospital doctors? a) Not at all accurate b) Slightly accurate c) Averagely accurate d) Fairly accurate e) Very accurate
8. How accurate is the information you receive from general practitioners? a) Not at all accurate b) Slightly accurate c) Averagely accurate d) Fairly accurate e) Very accurate
9. How accurate is the information you receive from the Sprengel nursing staff? a) Not at all accurate b) Slightly accurate c) Averagely accurate d) Fairly accurate) Very accurate
10. To what extent is communication with hospital doctors solution oriented (i.e., focused on solving problems rather than blaming)? a) Not at all solution oriented b) Slightly solution oriented c) Averagely solution oriented d) Fairly solution oriented e) Very solution oriented
11. To what extent is communication with general practitioners solution oriented (i.e., focused on solving problems rather than blaming)? a) Not at all solution oriented b) Slightly solution oriented c) Averagely solution oriented d) Fairly solution oriented e) Very solution oriented
12. To what extent is communication with the district's nursing staff solution oriented (i.e., focused on solving problems rather than blaming)? a) Not at all solution oriented b) Slightly solution oriented c) Averagely solution oriented d) Fairly solution oriented e) Very solution oriented
13. To what extent do you share common goals with hospital doctors? a) Not at all b) A little c) Partly/Sometimes d) Quite a bit e) Completely
14. To what extent do you share common goals with general practitioners? a) Not at all b) A little c) Partly/Sometimes d) Quite a bit e) Completely
15. To what extent do you share common goals with the Sprengel nursing staff? a) Not at all b) A little c) Partly/Sometimes d) Quite a bit e) Completely

16. To what extent do you have common knowledge about the respective tasks and responsibilities of hospital doctors? a) Not at all b) A little c) Partly/Sometimes d) Quite a bit e) Completely
17. To what extent do you have common knowledge about the respective tasks and responsibilities of general practitioners? a) Not at all b) A little c) Partly/Sometimes d) Quite a bit e) Completely
18. To what extent do you have common knowledge about the respective tasks and responsibilities of the Sprengel nursing staff? a) Not at all b) A little c) Partly/Sometimes d) Quite a bit e) Completely
19. To what extent do you have mutual respect in your collaboration with hospital doctors? a) Not at all b) A little c) Partly/Sometimes d) Quite a bit e) Completely
20. To what extent do you have mutual respect in your collaboration with general practitioners? a) Not at all b) A little c) Partly/sometimes d) Quite a bit e) Completely
21. To what extent do you have mutual respect in your collaboration with the Sprengel nursing staff? a) Not at all b) Little c) Partly/Sometimes d) Quite a bit e) Completely

Part 2—Job Satisfaction and Intention to Stay

22. Overall, how satisfied are you with your current job? a) Very dissatisfied b) Dissatisfied c) Averagely satisfied d) Fairly satisfied) Completely satisfied
23. "I plan to leave the South Tyrolean medical company/the convention with the country as soon as possible." a) Completely agree b) Somewhat agree c) Neither agree nor agree d) Somewhat disagree e) Strongly disagree to

Part 3—Working Conditions

24. How do you rate the quality of the current communication between health authorities/administration and general practitioners? a) Very good b) Good c) Sufficient d) Poor
25. How important is regular and effective communication with general practitioners to you? a) Very important b) Important c) Less important d) Not important
26. Which main communication channels do you currently use to pass on information to general practitioners and exchange information with them? a) Circulars b) E-mails c) Telephone calls d) Other (please specify) ________________________
27. What improvements do you see as urgent in terms of communication between health authorities/administration and general practitioners?) Establishment of a central contact point d) Greater involvement of general practitioners in decisions e) Other (please specify)

    ____
28. How do you rate the current bureaucratic burden on general practitioners in connection with administrative health services such as the application of ticket exemptions and AIFA grades as well as the issuance/extension of therapy plans? a) very high b) high c) medium d) low
29. How do you rate the current bureaucratic burden on hospital doctors in connection with administrative health services such as the application of ticket exemptions and AIFA grades as well as the issuance/extension of therapy plans? a) very high b) high c) medium d) low
30. Would you support the creation of general practice pathways to simplify the referral of patients for specialist healthcare services? a) Yes, without restrictions b) Yes, but only under certain conditions c) No, I see no need for it d) No opinion on it
31. How important do you think it is to improve communication and coordination of collaboration between patients, general practitioners, specialists and authorities to reduce inappropriate specialist referrals? a) Very important b) Important c) Less important d) Unimportant

32. How do you assess the current situation regarding questionably appropriate specialist assignments? a) Very good b) Good c) Bad d) Very bad
33. What role do you play in the healthcare system? Open question

    ____

34. Should patients be better informed about the importance of the gatekeeper role of general practitioners and the priority system for referrals? Open question

    ____

35. What roles do technological innovations play in improving the situation? Open question

    ____

Part 4—Additional Questions

- Sociodemographic information (age, gender, health district)
- How long have you been working in the health authority or in the healthcare system?
- What tasks and responsibilities do you have within the health authority or in the healthcare system?

Questions for out-patient nursing staff

Part 1—Relational Coordination Survey

1. How often do you communicate with general practitioners? a) Never b) Rarely c) Sometimes d) Frequently e) Always
2. How frequently do you communicate with administrative staff? a) Never b) Rarely c) Sometimes d) Frequently e) Always
3. How promptly does communication with general practitioners take place? a) Very delayed b) Delayed c) Average d) Timely e) Very timely
4. How timely is communication with administrative staff? a) Very delayed b) Delayed c) Averaged) Timely e) Very timely
5. How accurate is the information you receive from general practitioners? a) Not at all accurate b) Slightly accurate c) Averagely accurate d) Fairly accurate e) Very accurate
6. How accurate is the information you receive from administrative staff? a) Not at all accurate b) Slightly accurate c) Averagely accurate d) Fairly accurate) Very accurate
7. To what extent is communication with general practitioners solution oriented (i.e., focused on solving problems rather than blaming)? a) Not at all solution oriented b) Slightly solution oriented c) Averagely solution oriented d) Fairly solution oriented e) Very solution oriented
8. To what extent is communication with administrative staff solution oriented (i.e., focused on solving problems rather than blaming)?a) Not at all solution oriented b) Slightly solution oriented c) Averagely solution oriented d) Fairly solution oriented e) Very solution oriented
9. To what extent do you share common goals with general practitioners? a) Not at all b) A little c) Partly/Sometimes d) Quite a bit e) Completely
10. To what extent do you share common goals with the administrative staff? a) Not at all b) A little c) Partly/Sometimes d) Quite a bit e) Completely
11. To what extent do you have common knowledge about the respective tasks and responsibilities of general practitioners? a) Not at all b) A little c) Partly/Sometimes d) Quite a bit e) Completely

12. To what extent do you have common knowledge about the respective tasks and responsibilities of the administrative staff? a) Not at all b) A little c) Partly/Sometimes d) Quite a bit e) Completely
13. To what extent do you have mutual respect in your collaboration with general practitioners? a) Not at all b) A little c) Partly/sometimes d) Quite a bit e) Completely
14. To what extent do you have mutual respect in your cooperation with the administrative staff? a) Not at all b) A little c) Partly/Sometimes d) Quite a bit e) Completely

Part 2—Job Satisfaction and Intention to Stay

15. Overall, how satisfied are you with your current job? a) Very dissatisfied b) Dissatisfied c) Averagely satisfied d) Fairly satisfied) Completely satisfied
16. "I plan to leave the South Tyrolean medical company/the convention with the country as soon as possible." a) Completely agree b) Somewhat agree c) Neither agree nor agree d) Somewhat disagree e) Strongly disagree to

Part 3—Working Conditions

17. How satisfied are you generally with the collaboration and communication between you and the family doctors? a) Very satisfied b) Satisfied c) Dissatisfied d) Very dissatisfied
18. If anything is unclear, can you reach your family doctor by phone? a) Frequently b) Occasionally c) Rare d) Never
19. How often do you contact family doctors via email about patients? a) Frequently b) Occasionally c) Rarely d) Never
20. Do you have any further comments or suggestions for improving collaboration and communication with family doctors? (Open question)

Part 4—Additional Questions

- Sociodemographic information (age, gender, health district, city/rural or population categories of the place)
- How many years have you worked in the nursing service?
